# The Importance of Ear Canal Microbiota and Earwax in the Prevention of Outer Ear Infections

**DOI:** 10.3390/ijms27020622

**Published:** 2026-01-08

**Authors:** Paulina Paprocka, Jakub Spałek, Tamara Daniluk, Szczepan Kaliniak, Bonita Durnaś, Sławomir Okła, Robert Bucki

**Affiliations:** 1 Department of Microbiology and Immunology, Institute of Medical Science, Collegium Medicum, Jan Kochanowski University, IX Wieków Kielc 19A, 25-317 Kielce, Poland; paulina.paprocka@ujk.edu.pl; 2 Holy-Cross Cancer Center, Artwińskiego 3, 25-734 Kielce, Poland; k.spa@wp.pl (J.S.); szczepan.kaliniak@gmail.com (S.K.); bonita.durnas@ujk.edu.pl (B.D.); slawekok@gmail.com (S.O.); 3 Institute of Medical Science, Collegium Medicum, Jan Kochanowski University, IX Wieków Kielce 19A, 25-317 Kielce, Poland; 4 Department of Medical Microbiology and Nanobiomedical Engineering, Medical University of Białystok, 15-089 Białystok, Poland; tamara.daniluk@umb.edu.pl

**Keywords:** ear canal, cerumen, antibacterial peptides, endogenous lipid

## Abstract

This article describes the microbiome of the outer ear and the earwax in the ear canal, which performs various protective functions against bacterial infections. This article is based on an analysis of literature gathered from databases including PubMed, Google Scholar, Web of Science, and Scopus, primarily from the last 15 years. The search strategy included MeSH terms: ear canal, microbiome, earwax, cerumen, antibacterial peptides, ear infections, biofilm. Only peer-reviewed articles were included. The natural ear canal microbiota provides so-called colonization resistance, which protects against invasion by pathogenic microorganisms. Earwax is composed primarily of keratin secreted by epithelial cells and substances secreted by sweat and apocrine glands. It plays a key role in the physiology of the ear canal, maintaining a low pH, limiting moisture, and exhibiting antimicrobial properties. Both an excess and a deficiency of earwax can lead to dysbiosis of the outer ear, and consequently to the development of various infections. In an era of increasing antibiotic resistance and the search for new solutions in the fight against pathogenic microorganisms, understanding the natural properties of earwax is becoming increasingly important.

## 1. Introduction

External ear infection (OE) is a common laryngological infection. It affects 1–10% of the population and most often occurs in children and adolescents. The most common etiological factors of OE include: *Pseudomonas aeruginosa* and *Staphylococcus aureus* [1,2,3,4]. Less frequently isolated bacteria include: *Proteus mirabilis*, *Klebsiella* spp. *Escherichia coli* [5] and fungi: *Candida albicans*, *Candida parapsilosis* and *Aspergillus* spp. [6]. Some viruses, such as varicella-zoster (VZV), might also cause ear infections [7,8,9]. OE most often manifests itself with swelling, itching, pain and discharge. According to some reports its most common cause is moisture and improper ear cleaning methods [10], as well as the use of hearing aids [11] According to Sjövall et al., long-term use of hearing aids reduces the diversity of bacteria in the ear canal microbiota, leading to dysbiosis and more frequent infections [12]. Patients predisposed to this infection include those who suffer from atopic dermatitis, psoriasis, or have been diagnosed with anatomical defects and mechanical injuries [13].

**Figure 1:**
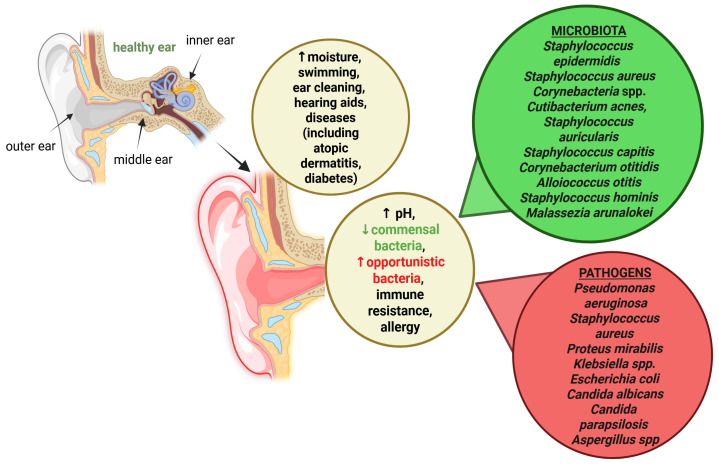
A schematic summary of factors that increase the risk of outer ear infections (otitis externa), highlighting environmental factors (e.g., water exposure and humidity), mechanical damage to the ear canal epithelium, disturbances in cerumen composition, and microbial imbalance, all of which contribute to impaired local immune defense.

Earwax in the outer ear is removed naturally. For most people, mechanical removal is not necessary. It is recommended for older people, hearing aid wearers, or those with excessive earwax production. Excessive earwax can cause tinnitus, hearing loss, ear congestion, and promote infections. Conversely, its deficiency increases the risk of infection by disrupting the microbiome Figure 1 [14].

## 2. Outer Ear Microbiome

When analyzing the composition of the skin microbiome, it can be observed that it differs depending on the area being examined. The composition of the ear skin microbiome has not yet been thoroughly studied but the research indicates that the diversity of microorganisms in a healthy ear was greater than in the ear in which the infection occurred [15]. In addition to the analysis of the organic components of earwax, it is also worth paying attention to the difference in the composition of the outer ear microbiota [16].

During the preparation of this manuscript, we analyzed many studies comparing the composition of the ear canal microbiome of healthy individuals with samples collected from patients with otitis externa. A combined analysis of these studies showed that the ear canal microbiome of healthy individuals consists primarily of *Staphylococcus epidermidis*, *Staphylococcus aureus*, and *Corynebacteria* spp. [16,17], *Cutibacterium acnes*, *Staphylococcus auricularis*, *Staphylococcus capitis/caprae*, *Corynebacterium otitidis*, *Alloiococcus otitis*, *Staphylococcus hominis* [3] *Neisseria* spp. and *Corynebacterium* spp. [18]. Of the fungi, the most numerous were *Malassezia arunalokei*, *Malassezia resticta*, and *Malassezia globosa* [3,18]. On the other hand, in the analyzed articles concerning patients with external otitis from whom ear swabs were taken, it was found that the most frequently occurring bacterium was *Pseudomonas aeruginosa*, followed by *Cutibacterium acnes*, *Staphylococcus aureus*, *Corynebacterium jeikeium*, *Klebsiella aerogenes/pneumoniae*, *Proteus mirabilis* and *Parvimonas* spp. Regarding fungal species, these included *Aspergillus* spp., *Malassezia restricta*, *Mallassezia arunalokei*, and *Candida albicans* [3,13,18,19]. Differences in the composition of the microbiome were also noted, which may vary between patients depending on age and gender [20,21].

The etiological factors responsible for otitis externa are also present in the ear microbiota of healthy individuals, but in smaller quantities. This means that the disease is caused not only by their presence but also by changes in the composition of the entire ear microbiome [22]. Interestingly, according to research by Amai et al. [20], genetic polymorphism might also determine the composition of the outer ear microbiota.

## 3. Comparison of the Ear Microbiome Composition in Healthy Individuals and Patients with External Ear Infections

When analyzing the composition of the skin microbiome, we can see that it varies depending on the site being examined. In wet and dry places on our body, we distinguish different types of microbes [15]. The human skin microbiota consists of bacteria, fungi, and viruses. The microorganisms that make up the human skin microbiota interact with each other, and their numbers can change depending on the environment in which a person lives [18]. The composition of the ear skin microbiome has not yet been thoroughly studied, but the research of Kim et al. indicates that the quantity of microorganisms in a healthy ear was greater than in the ear in which the infection occurred. They also confirm the fact that disorders in the composition of the microbiome contribute to the occurrence of various diseases. The study of the composition of the human microbiome provides many interesting results and proves that the occurrence of many diseases is related to the composition of the microbiome. That is why the number of studies in this field is constantly increasing [15].

**Figure 2:**
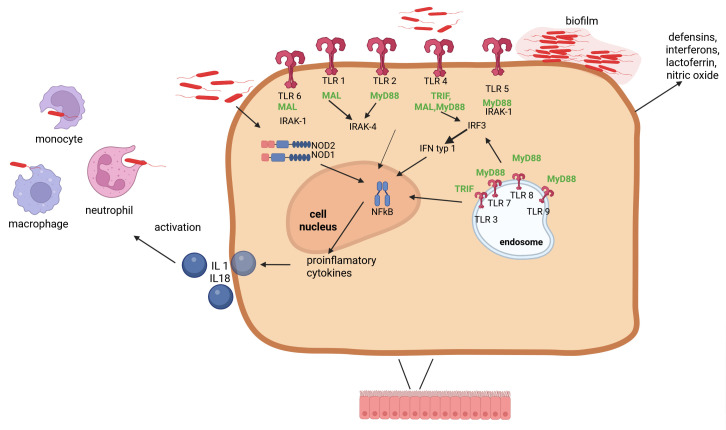
Bacteria colonizing the skin of the ear canal influence the non-specific response of immunocompetent cells present in this area (neutrophils, macrophages), and modulate epithelial cell responses, increasing the production of some interleukins and modulating signaling pathways involving TLR receptors.

To understand how bacteria can contribute to the development of outer ear infections, it is important to consider their virulence factors. Many types of bacteria that cause outer ear infections have the ability to form biofilms, which increase their chances of survival due to resistance to antibiotics, pH changes, and differences in humidity and temperature [23]. Other bacteria found in the ear canal microbiota, including *Staphylococcus* spp., can penetrate deeper layers of the skin and cause infection when the skin is scratched. Thanks to the alpha toxin they secrete, they damage tissue. Another type of bacteria found in the ear canal microbiota is *Corynebacterium* spp., which not only causes inflammation but can also regulate the expression of TLR2 and the proinflammatory proteins IL-1β, IL-6, and CSF3. Pathogens such as bacteria from the genera *Pseudomonas* spp. and *Acinetobacter* spp. also produce toxins that can contribute to tissue damage. Their high resistance to antibiotics and the host’s immune system allows them to easily colonize and cause infection [21,24,25,26], Figure 2.

According to a study by Burton et al., of 92 ear swabs collected from healthy individuals, *Cutibacterium acnes* was isolated in every sample and in the largest quantity. Smaller quantities included *Staphylococcus auricularis*, *Staphylococcus capitis/caprae*, *Corynebacterium otitidis*, *Alloiococcus otitis*, *Staphylococcus hominis*, and *Staphylococcus epidermidis*. As for fungal species, the largest quantities were *Malassezia arunalokei*, *Malassezia resticta*, and *Malassezia globosa*. On the other hand, in ear swabs collected from individuals with otitis externa, the largest quantity was *Peudomonas aeruginosa*, followed by, among others. *Cutibacterium acnes*, *Staphylococcus aureus*, *Corynebacterium jeikeium*, *Klebsiella aerogenes/pneumoniae*, and *Proteus mirabilis*. As for the species of fungi that occurred in the diseased ear, these included *Aspergillus* spp., *Malassezia restricta*, *M. arunalokei*, and *Candida albicans*. These researchers also confirmed that the microbiota of the healthy ear is more diverse compared to the ear with ongoing inflammation [3].

Other studies conducted by Lee et al. on 24 patients with chronic otitis media and 24 healthy individuals showed that in both healthy and sick individuals, the ear canal microbiome was composed mostly of bacteria from the *Staphylococcus* spp. and *Cutibacterium* spp. genera. In healthy individuals, additional bacteria from the *Neisseria* spp. and *Corynebacterium* spp. genera were isolated. In terms of fungi, the largest number in healthy individuals was *Malassezia restricta*, while in sick individuals, it was *Aspergillus niger* and *Candida albicans*. This confirms the fact that the latter are the most common species of fungi causing ear infections [18].

In another study, Sjövall et al. examined 41 external ear canal swab samples from people without signs of infection and found that the highest number of bacteria in the healthy ear were also *Staphylococcus auricularis*, *Cutibacterium acnes*, *Alloiococcus otitis*, and *Cutibacterium otitidis* [27]. As various authors point out, differences in the composition of the skin microbiota in the ear may result from different patient ages, genders, and races, which indicates different types of earwax, or environmental factors [18,27].

Alhussaini et al., examining a total of 174 samples taken from patients (159) and (15) healthy individuals, drew attention to the fact that the composition of microorganisms included in the microbiota of the ear canal in people using hearing aids differs from that of healthy individuals. In most people, the infection was caused by bacteria such as coagulase-negative *Staphylococci* (CNS), *Staphylococcus aureus* and *Pseudomonas aeruginosa*, while infections caused by *Candida albicans* fungi were less common. The authors also noted that the type of hearing aid used by patients is important for the occurrence of a given etiological factor. Namely, patients using Complete in the canal (CIC) hearing aids are much more susceptible to infections caused by *Candida albicans* compared to patients using Behind the ear (BTE). However, when analyzing infections caused by a bacterial etiological factor, it is not so clear Table 1 [19].

The external auditory canal is about 2.5 cm long, and its microbiota includes microorganisms that constitute the skin microbiota. As Klein emphasizes, these are mostly *Staphylococcus epidermidis*, *Staphylococcus aureus*, *Corynebacteria* spp. A smaller number are anaerobic bacteria such as *Cutibacterium acnes* [16]. Most of the bacteria naturally occurring in the external ear canal are Gram-positive bacteria. They can constitute up to 90% of all microorganisms found there. Their number can change depending on the pH of the ear canal, the composition of the cerumen, as well as the dysfunction of the immune system [31]. Research indicates that ear infection, excessive moisture, and sweating contribute to an increase in the pH of the ear canal, which increases susceptibility to infections with pathogenic microorganisms. Therefore, antibiotics and corticosteroids are most often used during treatment, in combination with acidifying medications, to lower the pH and accelerate healing [32,33].

As it results from the above studies, microorganisms included in the microbiota of the external ear were also isolated from patients without signs of disease. The etiological factors responsible for otitis externa occur in the microbiota of healthy people, but in smaller quantities. This means that the occurrence of the disease is not only caused by their presence, but also by a change in the composition of the entire microbiome of the ear [21,22].

Otitis externa can sometimes be a cause or consequence of certain diseases. The outer ear is the most common site for foreign objects in young children, which can contribute to inflammation, eventually spreading to the middle and inner ear. In some cases, trauma can also contribute to perichondritis. Another condition associated with external ear infection is otomycosis, which can occur in the outer ear and may be associated with an acute, subacute, or chronic fungal infection (caused by molds, yeast-like fungi, and in patients with untreated dermatomycosis), or may be the result of bacterial infection and the use of topical medications. Sometimes, keratinization and the accumulation of large amounts of keratin (a component of cerumen) can lead to obstructive keratinization, which can ultimately contribute to the development of primary cholesteatoma of the auditory canal [34,35]. Eczematous otitis externa, according to research by Celebi et al., is more common in patients struggling with allergies. On the one hand, it may be the first sign that these individuals should undergo allergy testing, but on the other hand, it is important to remember that it may be more common in allergy sufferers [36].

## 4. Composition of Earwax

### 4.1. Cerumen Composition and Role

The substance that provides natural protection to the ear canal is cerumen, commonly known as earwax. The composition of cerumen has been analyzed since 1953 [37]. Cerumen is composed of the secretion of sebaceous glands and apocrine glands and epithelial debris [38]. Earwax acidifies the environment in the ear, thus protecting it from bacteria and fungi [39]. Sometimes, when its composition changes, it can lead to the development of an OE, frequently caused by *Pseudomonas aeruginosa* and *Staphylococcus aureus* [1,2,3]. Other microorganisms such as *Haemophilus influenzae*, *Proteus mirabilis*, *Klebsiella* spp. *Escherichia coli*, *Streptococcus pneumoniae* [5] *Candida albicans*, *Candida parapsilosis* and *Aspergillus* spp. might also cause ear infections but less frequently [6,7,8,9].

### 4.2. Different Types of Earwax and Their Associated Susceptibility to Disease

There are two types of earwax. The first is brittle, dry earwax characterized by yellowish and grayish color, most often occurring in people of Asian and Native American origin, and wet type earwax, which is sticky and slippery, dominant in people of African and European origin [40,41]. The type of cerumen is determined by the *ABCC11* gene located on chromosome 16 [42]. The 538G>A single-nucleotide polymorphism (SNP) in this gene determines the role of cerumen. *ABCC11* (538G and 538GA) are responsible for wet cerumen, while *ABCC11* (538AA) determines dry cerumen [20,43,44].

Scientists have discovered that a single DNA sequence change in the *ABCC11* gene may be responsible for the development of dry earwax. Furthermore, mutations in the *ABCC11* gene reduce lipid transport across cell membranes and the function of ATP-binding cassette (ABC) transporters, which are mediated by the MRP8 protein. Furthermore, these mutations may affect the expression and activity of the MRP1 protein, which plays a key role in the inflammatory response. These processes are crucial for the proper secretion and removal of cerumen and contribute to maintaining a healthy ear [42].

It has been observed that individuals with wet earwax are more likely to have conditions such as axillary osmidrosis and middle ear cholesteatoma. Other conditions, such as otosclerosis, ear canal cancer, and age-related hearing loss, are gender-related and occur more frequently in women. Interestingly, the number of *Streptococcus* spp. in the ear canal is significantly higher in women. Differences can also be observed in infants—Caucasians have a higher incidence of acute otitis media than Asians [20]. In general, scientists have noted that people with dry earwax are less prone to ear infections than those with wet earwax [42].

Cerumen is a hydrophobic substance that, thanks to the movements of the cilia on the epithelium, can move along the ear canal and clean it. Interfering with the natural self-cleaning process of the outer ear by using cotton buds or hearing aids causes earwax to be pushed deeper into the ear canal, preventing it from being cleaned properly. Another problem may be the increased production of cerumen in relation to the speed of its removal from the ear canal. This may explain the fact that in old age, when the hair in the ear canal is thicker, the speed of cerumen removal is slower, which in turn may explain the more frequent accumulation of earwax in this group of patients [40].

**Figure 3:**
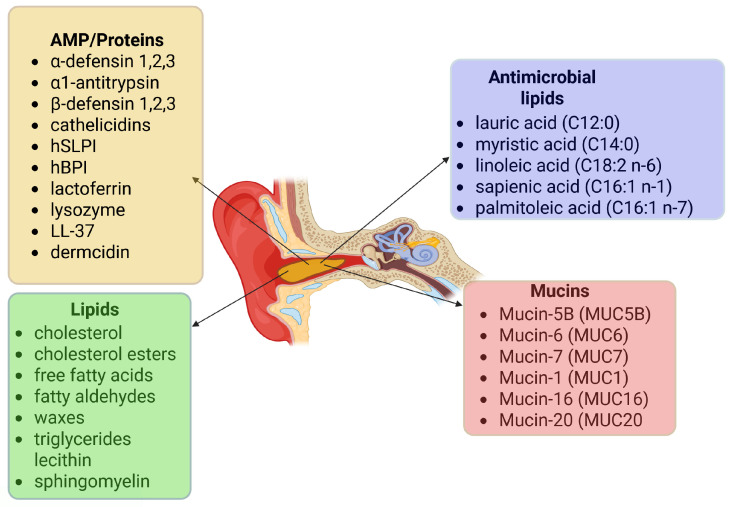
Cerumen composition includes four major groups of compounds: mucins, lipids, antimicrobial lipids, and antimicrobial peptides (AMPs)/proteins. These components collectively maintain moisture, trap particulate matter, and provide protection against microbial colonization in the external auditory canal.

Earwax contains lipids, mucins, antimicrobial peptides, proteins, sphingosines and two types of immunoglobulins IgG and IgA, thanks to which the ear canal is effectively protected against infections. These substances have a wide spectrum of action against various microorganisms, including bacteria, fungi, viruses and protozoa Figure 3 [38,45].

### 4.3. Antimicrobial Potential of Earwax

**Figure 4:**
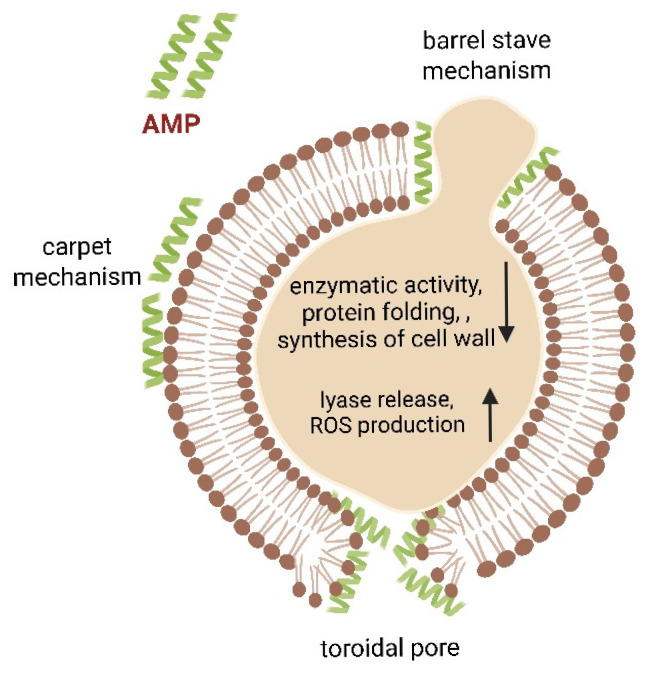
Schematic representation of the mechanism of action of antimicrobial peptides (AMPs). AMPs interact with and insert into the microbial cell membrane, causing disruption of the lipid bilayer structure, increased membrane permeability, leakage of intracellular contents, and loss of the membrane’s ability to regulate molecular exchange between the cytosol and the extracellular environment.

One of the ingredients naturally produced by our immune system to prevent infections caused by microorganisms are antimicrobial peptides (AMPs). AMPs, as molecules with a positive charge, have a high affinity for molecules facing the external environment of both Gram-positive and Gram-negative bacteria. Their mechanism of antimicrobial action may vary depending on their structure, but they typically increase permeabilization and destruction of the cell membrane upon charge driving interaction with negative moieties of the bacterial membrane. Additionally, they may inhibit protein synthesis. There are several described mechanisms leading to membrane damage upon antibacterial peptides attack. Different models including “barrel stave”, “carpet mechanism” and “toroidal pore” were proposed to described they mechanism of action. So far, microorganisms are less likely to develop resistance to AMPs, so these peptides are a good alternative in the treatment of infections caused by resistant strains of microorganism Figure 4 [46,47,48,49,50,51,52,53].

**Figure 5:**
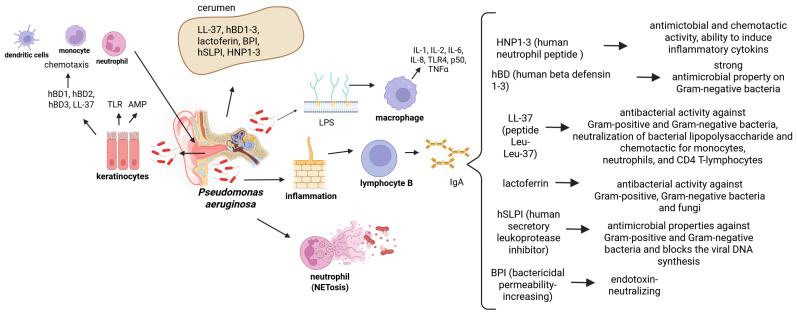
A schematic diagram illustrating the activation of the inflammatory process in the outer ear caused by the bacterium *Pseudomonas aeruginosa*.

In human earwax, many AMPs have been identified that protect the auditory ear canal from infections. These include human beta defensins (hBD 1-3), which have strong antibacterial effects on various microorganisms, including *Escherichia coli*, *Pseudomonas aeruginosa*, *Staphylococcus aureus*, *Streptococcus pyogenes*, *Haemophilus influenzae*, *Enterococcus faecium* and *Candida albicans*, human cathelicidin protein LL-37 which exhibits chemotactic activity on immunocompetent cells and antibacterial activity against Gram-positive and Gram-negative bacteria. It also neutralizes bacterial lipopolysaccharide. Another important component of earwax is human lactoferrin (Lfc). It has antibacterial activity against *Streptococcus mutans*, *Vibrio cholera*, *Escherichia coli*, *Actinobicillus actinocetemitityans*, *Legionella pneumophila*, *Pseudomonas aeruginosa* and *Candida albicans*. Earwax also contains human secretin, a leukoprotease inhibitor (hSLPI), found on the surfaces of epithelial cells, macrophages and neutrophils, which has antibacterial properties against Gram-positive and Gram-negative bacteria and also blocks viral replication [54,55]. Cerumen also contains human BPI protein, found in neutrophil granules and skin fibroblasts. Another identified protein is human neutrophil peptide (HNP1-3), found in neutrophil granulocytes, involved in regulating inflammation, complement activation, cytotoxicity, and chemotaxis of immune system cells (Figure 5) [24,25,26,55]. It is also noted that the use of AMP in conjunction with antibiotics increases the effectiveness of treatment and helps combat antibiotic-resistant pathogens. By disrupting the integrity of bacterial membranes, AMP helps deliver the antibiotic into the bacterial cell, thereby enhancing its effectiveness [56].

In addition to AMPs, nitric oxide peroxidase, complement proteins, enzymes secreted by keratinocytes and skin glands, and endogenous lipid molecules (AMLs) have recently been described in cerumen [57]. The lipids that make up cerumen include: triacylglycerols free fatty acids, cholesterol, including cholesterol esters and sulphates [58]. All of these also exhibit antimicrobial activity. After entering the cytoplasm, some of them can inhibit the action of cytosolic enzymes and the synthesis of bacterial fatty acids. Once bound to the bacterial cell surface, they can change the physical properties of the membrane, such as its fluidity, and also disrupt ion transport. Little is known about the action of antimicrobial lipids but it has been noted that the cell membrane is their main target. Their action consists of preventing water loss and hindering the penetration of microorganisms and their toxins into the human body. An example of such lipids found in the skin, among others, are sphingoids. They act on Gram-negative and Gram-positive bacteria, viruses and fungi. The action of sphingoids is to inhibit the synthesis of molecules that build bacterial cell walls. Some studies also indicate the effective use of AMLs as substances preventing the formation of biofilm. When it comes to triglycerides produced by sweat glands in the skin, lauric acid and sapienic acid show the best antimicrobial effects. Some AMLs are already used in production, including undecylenic acid as an antifungal agent added to denture inserts to prevent the growth of *Candida albicans*, or used in patients infected with herpes simplex viruses to reduce pain and virus replication. Lauric acid is also used in the treatment of acne. In the case of AMLs, a similar effect is observed depending on the dose used, as in the case of AMPs [57].

AMPs and AMLs are compounds that have antibacterial activity both when used alone and in combination. Studies indicate that the combination of several AMPs, such as indolicidin, LL-37 and bactenecin, exhibits synergistic activity against *Pseudomonas aeruginosa* and *Escherichia coli* [56]. In line with previous studies, AMLs also show synergistic effects both when combining two different AMLs, such as phytosphingosine and N-lauryl arginine ethyl ester laurate, and with AMP (sphingoid with cathelicidin LL-37). Lauric acid has also been shown to enhance chlorhexidine activity against *S. mutans.* AMPs and AMLs are unlikely to cause side effects or develop microbial resistance, so research is underway to better characterize these molecules through translation for potential use in future ear infection treatments [57].

## 5. Treatment and Prevention of Otitis Externa

Treatment of inflammation in the ear should begin with cleaning the ear canal of cerumen, which may contain microorganisms and their products. This treatment is used to reduce inflammation and increase the effectiveness of local medications, the absorption of which is limited in the case of a clogged ear canal. In the first stage, it is not recommended to administer topical drops because they may be irritating. The next step is to administer painkillers and local medications, and if the symptoms do not subside after 48–72 h or in the case of acute diffuse otitis externa (OEAD), especially in patients with diabetes and during immunosuppressive treatment, general antibiotic therapy is recommended [1,59]. Antibiotics should not be used in the first phase of the disease due to multidrug resistance of microorganisms, the possibility of side effects, as well as the clinical condition of patients [9].

Treatment of otitis in patients is mainly based on the administration of anti-inflammatory and analgesic drugs. Local antibiotics, which achieve the highest concentration at the target site, are also used successfully. Antibiotics used to treat this type of infection include aminoglycosides (gentamicin and neomycin), polymyxin B, or fluoroquinolones (ciprofloxacin) [9,60]. It is also recommended to use antibiotics combined with a steroid, which reduces inflammation and shortens the treatment time, as well as antiseptics with a low pH. There is no specific group of antibiotics indicated for the treatment of OE, but ciprofloxacin is an antibiotic that can also be used in patients with a perforated eardrum [1]. In this group of patients, it is also recommended to use drops with neomycin, polymyxin B, hydrocortisone, aminoglycosides, and alcohols [39]. For topical use, preparations containing, e.g., acetic acid, boric acid and liposomal ozone are recommended Table 2 [13].

Special problems are caused by the treatment of OE caused by several types of bacteria, several types of fungi or mixed bacteria and fungi, or aerobic and anaerobic bacteria at the same time. The choice of treatment in such cases is difficult. Due to the increasing incidence of infections whose etiological factors are many different microorganisms at the same time, some researchers recommend introducing changes in the method of treatment. In addition to administering antibacterial antibiotics to patients, it is also proposed to administer antifungal substances [65]. On the other hand, long-term use of antibiotics also contributes to the occurrence of fungal infections [66].

Cerumen removal occurs naturally, but if the patient suffers from ear canal blockage caused by excessive cerumen secretion, it is recommended to use water-based preparations (5% potassium hydroxide, hydrogen peroxide, acetic acid, sodium bicarbonate), oils (peanut oil, olive oil, and almond oil) and carbamide peroxide. The use of water-based preparations is intended to moisturize the cerumen and facilitate its removal, preparations that contain oils exfoliate and soften the earwax, while those with carbamide peroxide also soften cerumen and have an antibacterial effect. All of these substances are commonly used by general practitioners and otolaryngologists to treat ear canal blockage [67,68,69].

Due to the high multidrug resistance of microorganisms causing otitis externa, as well as comorbidities of patients, therapeutic options are sometimes limited. New solutions for the use of drugs and new alternative substances are being sought to combat these infections. One such substance is nanocrystalline silver, which has anti-inflammatory, antibacterial and antiseptic properties. Silver has been used in medicine since 1880, but the development of nanotechnology has contributed to the construction of a molecule that has increased bioactivity and contributes to obtaining a better therapeutic effect [10]. Other substances tested are thyme essential oil or clove extract. They also constitute an alternative method of treatment, especially since they do not show the same side effects as drugs used in standard therapy, which is why they are increasingly used in treatment [70]. Recently, new liposomal ozone-based drugs have also been used, which also show anti-inflammatory and antiseptic effects. Ozone itself has an irritating effect on the ear mucous membranes, but the combination with liposomes makes it much more tolerable and has an inhibitory effect on bacteria and fungi, as well as an anti-biofilm effect [13].

More and more research is being carried out on substances that could be used to coat various medical materials such as catheters, dental implants or contact lenses. The antimicrobial effect of chemical compounds (TiO_2_, ZnO, Fe_2_O_3_, MgO, ZrO_2_), metal nanoparticles (Ag, Cu, Pt and Au) and analogs of natural peptides (AMP) ceragenins is gaining more and more supporters, and the results are satisfactory. However, there are no studies on the antibacterial effects of these compounds used as antibacterial coatings in earplugs used by swimmers [71,72,73].

However, there are studies confirming increased bactericidal activity of ceragenins in the presence of human cerumen. This is related to the presence of natural antibacterial peptides in cerumen. This in turn means that ceragenins in the presence of peptide LL-37 or lysozyme found in cerumen show synergistic effects. These results suggest that we should use these compounds as therapeutic agents in the treatment of ear infections [74].

This research could bring many benefits and, in the future, contribute to the introduction of earplugs to the market that prevent the development of etiological factors or the introduction of new substances that combat otitis externa.

## 6. Assessment of the Composition of Earwax and Its Use as a Diagnostic Material

The composition of earwax is diverse, and its function are not fully determined. Earwax contains, among others, keratin from exfoliated cells, which constitutes about 60% [68], saturated and unsaturated fatty acids, cholesterol, sebum secreted by sebaceous glands and secretion from sweat glands [75] and amino acids, neurostearic acid, cerotic acid, hexone bases, lysozyme, immunoglobulin (Ig), glycopeptides and copper [76]. According to a study conducted by Naz I., earwax also contains flavonoids and terpenoids, but it does not contain phenolic compounds. Some authors claim that earwax contains substances that promote the growth of microorganisms, while others concluded that due to the compounds contained in it and the acidic environment (pH 5.2–7.0) that occurs in the external ear thanks to them, it can effectively inhibit the growth of microorganisms and prevent infection [75,77,78,79]. Cerumen, which is found in the outer ear, is a very good hydrophobic barrier protecting the middle and inner ear from water penetration, damage and infections. It collects microorganisms, dirt, water and, thanks to oils and hairs on the epithelium, moves it to the outside of the ear canal while removing all dirt [76]. Earwax contains antimicrobial peptides, which also provide antibacterial protection. However, immunohistochemical studies confirm that it is not cerumen that is the main factor fighting infections at the time of infection, but the secreted immunoglobulins IgA and IgG. According to studies by Swain et al., human earwax has a greater antibacterial effect than antifungal effect.

### The Use of Earwax as a Diagnostic Material

Despite various studies that ambiguously determine the effect of earwax depending on the etiological factor, most researchers believe that it is a good barrier protecting against the penetration of microorganisms [76].

As various studies indicate, the assessment of the composition of earwax can also be used as a material for toxicological tests. In addition to urine and blood, which are standard materials in this type of research, the composition of hair, nails and sweat was also compared, which was compared to the composition of earwax. As it results from the studies, materials such as hair and nails have certain disadvantages due to the possibility of contamination from the external environment and contact with cosmetics. Sweat, on the other hand, due to the long time required for this measurement, is also not considered the best material. However, earwax, due to its high keratin content, like hair and nails, has an advantage over these materials. Easy sampling and reduced possibility of contamination make it a promising material used to determine the content of psychotropic drugs or narcotics in the patient’s body [80,81,82,83].

Other studies show that earwax can also be used as a material for testing sugar levels. The results obtained by Herane-Vives et al. and Shokry et al. prove that tests using earwax collected from the patients studied to determine sugar levels are more accurate than standard tests of glycosylated hemoglobin HbA1c. The method of collecting earwax was also much easier [84,85].

Shokry et al. also confirm the use of cerumen as a material that is a better option than blood, urine, sweat and hair, only this time for the study of the assessment of the amount of nicotine and its metabolites in active and passive smokers. As before, the use of cerumen is supported by the fact that it is a material obtained in an easy way and we can precisely monitor the amount of compounds to which the patient was exposed recently and even several months earlier [86].

According to the latest information, earwax is also used in diagnostics, among others. allergic rhinitis, otosclerosis, genetic diseases (maple syrup disease and alkaptonuria), as well as in the detection of breast cancer and some cancer biomarkers [87].

Although cerumen is an easy material to collect for clinical trials, it should be remembered that it is also a material that may contain the hepatitis B virus (HBV) and the SARS-CoV-2 virus. Therefore, it may be a source of infection with these viruses. Therefore, Hanege et al. emphasize the role of hygiene and the use of personal protective equipment when contacting this material [88].

## 7. Conclusions

A review of the literature confirms that the composition of earwax can vary, which may contribute to the increased incidence of outer ear infections in some individuals. The exact composition of earwax, the *ABCC11* gene polymorphism, and the relationships between its individual components have not yet been fully explored. Perhaps in the future, the results of this study will help us better understand what determines the incidence of outer ear infections and provide valuable information on the use of these components as new substances to support the treatment.

## Figures and Tables

**Table 1:** Microorganisms included in the microbiota of the skin of a healthy ear and those with external ear infection.

Microorganisms	Microbiota of the Ear Skin in a Healthy Person	Microbiota of the Ear Skin of a Person with an Outer Ear Infection
**Bacterials**	*Cutibacterium acnes*, *Muribaculum intestinale*, *Micrococcus aloeverae*, *Rothia mucilaginosa* [15], *Staphylococcus auricularis* [18], *Staphylococcus capitis. Staphylococcus caprae*, *Corynebacterium otitidis*, *Staphylococcus pettenkoferi*, *Staphylococcus hominis*, *Staphylococcus epidermidis*, *Cutibacterium humerusii*, *Streptococcus mitis*, *Streptococcus oralis*, *Streptococcus pneumoniae*, *Corynebacterium segmentosum*, *Corynebacterium pseudogenitalium*, *Corynebacterium tuberculostearicum* [3]	*Staphylococcus warneri Pseudomonas aeruginosa*, [15] *Staphylococcus pettenkoferi*, *Neisseriaceae* spp. [18], *Staphylococcus aureus* [19], *Cutibacterium acnes*, *Klebsiella aerogenes*, *Proteus mirabilis*, *Corynebacterium jeikeium*, *Corynebacterium otitidis*, *Staphyloccoocus caprae*, *Staphylococcus auricularis*, *Corynebacterium simulans* [3] *Enterococcus faecalis*, *Escherichia coli*, *Streptococcus pneumoniae*, *Serratia marcescens*, *Achromobacter xylosoxidans*, *Citrobacter koseri* [28], *Auritidibacter ignavus* [29]
**Fungi**	*Malassezia arunalokei*, *Malassezia restricta*, *Malassezia globosa*, *Malassezia slooffiae*, *Malassezia sympodialis*, *Candida metapsilosis/orthopsilosis*, *Alternaria* spp., *Cladosporium* spp., *Torula* spp., *Pleosporales* spp. [3]	*Malassezia restricta*, *Malassezia slooffiae*, *Malassezia globosa*, *Aspergillus niger*, *Candida albicans* [18], *Malassezia arunalokei*, *Candida parapsilosis*, *Aspergillus citrinoterreus/pseudoterreus/terreus*, *Cladosporium* spp., *Cryptococcus noformas*, *Penicillium* spp. [3], *Candida auris* [30]

**Table 2:** Etiological factors and drugs used to treat external ear infections.

Etiological Factor Causing Otitis Externa	Medicines Used to Treat Outer Ear Infections
*Pseudomonas aeruginosa*	Ciprofloxacin [9], Amikacin, Ceftazidime [61]
*Staphylococcus aureus*	Trimethoprim/sulfamethoxazole [9]
*Haemophilus influenzae*	Amoxicillin [62]
*Proteus mirabilis*	Gentamicin, amikacin [63]
*Escherichia coli*	Cefoxitin, ciprofloxacin [63]
*Streptococcus pneumoniae*	Amoxicillin [62]
*Candida albicans*, *Candida parapsilosis*, *Aspergillus* spp.	Nystatin, Iodoform, Pimafucin, Pimafucort, Ketoconazole, Orungal [64]

## Data Availability

No new data were created or analyzed in this study. Data sharing is not applicable to this article.

## Abbreviations

OE	external ear infection
VZV	varicella-zoster virus
CNS	coagulase-negative *Staphylococci*
CIC	complete in the canal hearing aids
BTE	behind-the-ear hearing aids
SNP	single-nucleotide polymorphism
AMPs	antimicrobial peptides
hBD 1-3	human beta defensins 1-3
Lfc	human lactoferrin
hSLPI	human leukoprotease inhibitor secretin
BPI	human protein
HNP1-3	human neutrophil peptide
AMLs	endogenous lipid molecules
LL-37	peptide Leu-Leu-37
OEAD	otitis externa acuta diffusa
HBV	hepatitis B virus
SARS-CoV2	severe acute respiratory syndrome coronavirus 2
